# What can neurofeedback and transcranial alternating current stimulation reveal about cross-frequency coupling?

**DOI:** 10.3389/fnins.2025.1465773

**Published:** 2025-02-12

**Authors:** Mária Orendáčová, Eugen Kvašňák

**Affiliations:** Department of Medical Biophysics and Medical Informatics, Third Faculty of Medicine, Charles University in Prague, Prague, Czechia

**Keywords:** EEG, cross-frequency coupling, neurofeedback, tACS, variability

## Abstract

In recent years, the dynamics and function of cross-frequency coupling (CFC) in electroencephalography (EEG) have emerged as a prevalent area of investigation within the research community. One possible approach in studying CFC is to utilize non-invasive neuromodulation methods such as transcranial alternating current stimulation (tACS) and neurofeedback (NFB). In this study, we address (1) the potential applicability of single and multifrequency tACS and NFB protocols in CFC research; (2) the prevalence of CFC types, such as phase–amplitude or amplitude–amplitude CFC, in tACS and NFB studies; and (3) factors that contribute to inter- and intraindividual variability in CFC and ways to address them potentially. Here we analyzed research studies on CFC, tACS, and neurofeedback. Based on current knowledge, CFC types have been reported in tACS and NFB studies. We hypothesize that direct and indirect effects of tACS and neurofeedback can induce CFC. Several variability factors such as health status, age, fatigue, personality traits, and eyes-closed (EC) vs. eyes-open (EO)condition may influence the CFC types. Modifying the duration of the tACS and neurofeedback intervention and selecting a specific demographic experimental group could reduce these sources of CFC variability. Neurofeedback and tACS appear to be promising tools for studying CFC.

## 1 Introduction

The study of electroencephalographic (EEG) activity related to brain function and its potential enhancement through neurotherapeutic tools has emerged as a significant area of research in neuroscience (Di Bernardi Luft et al., 2018; de la Salle et al., 2024). EEG bandwidths are classified into five basic categories: delta (1–4 Hz), theta (4–8 Hz), alpha (8–12 Hz), beta (13–30 Hz), and gamma (30 Hz and above; Buzsáki, 2006; Miller, 2007; Dressler et al., 2004). These EEG bandwidths have different functions. For example, occipital alpha is associated with visual processing (Klimesch, 1999). Frontal and parietal theta plays a significant role in working memory processes (Polanía et al., 2012). Beta activity is linked to attention (Groppe et al., 2013) and motor functions (Brignani et al., 2013). The EEG bandwidth can be further divided into finer subcategories based on the functions and neuroanatomical origins of the respective EEG frequencies within this bandwidth spectrum (Pfurtscheller et al., 1997; Klimesch, 1999). For example, lower alpha (approximately 8–10 Hz) is associated with the resting state, whereas higher alpha (approximately 10–12 Hz) is associated with cognitive tasks such as working memory processes (Klimesch, 1999).

However, a specific brain function seldom relies on just one type of EEG activity. Instead, several brain networks typically operate simultaneously at various EEG frequencies to facilitate complex brain functions (Lisman, 2010; Lisman and Jensen, 2013; Hyafil et al., 2015). For example, research has shown that not only alpha, as previously mentioned, but also gamma activity plays a significant role in visual functions, specifically in the visual representation of an object (Jensen et al., 2014). Similarly, independent studies have shown that working memory performance is positively associated with several EEG bandwidths, such as alpha (Escolano et al., 2012; Escolano et al., 2014), theta (Klimesch, 1999; Polanía et al., 2012), and gamma (Lisman, 2010). These findings have raised the question among researchers about whether and how multiple EEG frequencies interact with each other when they are involved in a common brain process simultaneously. The discovery of cross-frequency coupling (CFC), a phenomenon in which two or more different frequencies synchronize with each other (Jirsa and Müller, 2013; Yeh et al., 2023), answered this question. The biophysical and functional locking of different EEG frequency bandwidths to each other allows a more effective spatiotemporal information transfer between neuronal populations (Jirsa and Müller, 2013; Hyafil et al., 2015; Yeh et al., 2023). To date, numerous CFC patterns have been discovered, as well as their association with various brain functions such as sleep (Ngo et al., 2013; Ketz et al., 2018), memory (Popov et al., 2018; Biel et al., 2021), and emotional regulation (Bramson et al., 2020).

Although CFC is an intriguing area of research, the precise nature of neural processes beyond CFC and their connection to brain function remains elusive. The same applies to the relationship between specific frequency components of a given CFC pattern. For example, the close cooperation between alpha and theta appears to play a crucial role in memory processes (Popov et al., 2018). One may inquire: What will occur to theta during memory processes—an event in which both EEG bandwidths are significantly engaged—if we selectively stimulate alpha activity through an external stimulation at a corresponding frequency? Could we improve brain functions that depend on a particular CFC pattern by selectively entraining low and high-frequency CFC components?

Non-invasive brain modulation techniques that can interfere with ongoing EEG activity are a promising tool for addressing these questions. Non-invasive brain modulation includes modalities based on the periodic delivery of external acoustic (Ngo et al., 2013) and electromagnetic fields (Herrmann et al., 2013; Turi et al., 2020), which can modulate the amplitude and phase of the same or nearly the same ongoing EEG activity via entrainment (Liu et al., 2018). Transcranial alternating current stimulation (tACS) and neurofeedback (NFB) are two different non-invasive brain stimulation techniques that are widely used in neuroscience research.

The objectives of this paper are as follows:

1. To compare the potential applicability of single- and multifrequency tACS and NFB protocols in CFC research.
2. To discuss known CFC types and their occurrence in tACS and NFB studies.
3. Propose a further classification of CFC types based on the effects of tACS and NFB.
4. Discuss factors contributing to variability in CFC dynamics and potential ways to address them.

## 2 Materials and methods

An analysis of research articles related to tACS, NFB, and CFC was conducted. A search for articles was carried out using the PubMed database using keywords that included transcranial alternating current stimulation, neurofeedback, and cross-frequency coupling.

## 3 Mechanism of tACS

TACS requires two or more scalp electrodes immersed in a conductive medium to facilitate the flow of alternating current between the targeted brain areas (Herrmann et al., 2013). TACS represents external electric fields that are periodically induced and interfere with ongoing EEG activity (Antal and Herrmann, 2016; Antal and Paulus, 2013). Regarding the immediate effects of tACS on EEG—including tACS-induced changes in amplitudes and coherences of EEG activity, also referred to as online effects (Bland and Sale, 2019)—entrainment is considered to be the primary mechanism responsible for enhancing the phase alignment of the endogenous EEG frequency with the external tACS frequency (Antal and Paulus, 2013; Liu et al., 2018). This process leads to an increase in amplitude and/or coherence of the respective EEG frequency (Zaehle et al., 2010; Berger et al., 2018). Entrainment is the phenomenon that occurs when EEG activity with a frequency similar to that of tACS becomes phase-locked to the external driving tACS frequency (Liu et al., 2018; Krause et al., 2019). Regarding aftereffects (also referred to as offline effects of tACS) following tACS intervention, several studies have reported long-term aftereffects such as enhanced EEG amplitudes and coherence (Zaehle et al., 2010; Kasten and Herrmann, 2017; Ketz et al., 2018). The proposed underlying mechanism is believed to be brain plasticity (Vossen et al., 2015; Wischnewski et al., 2019).

## 4 Mechanism of NFB

**Figure 1:**
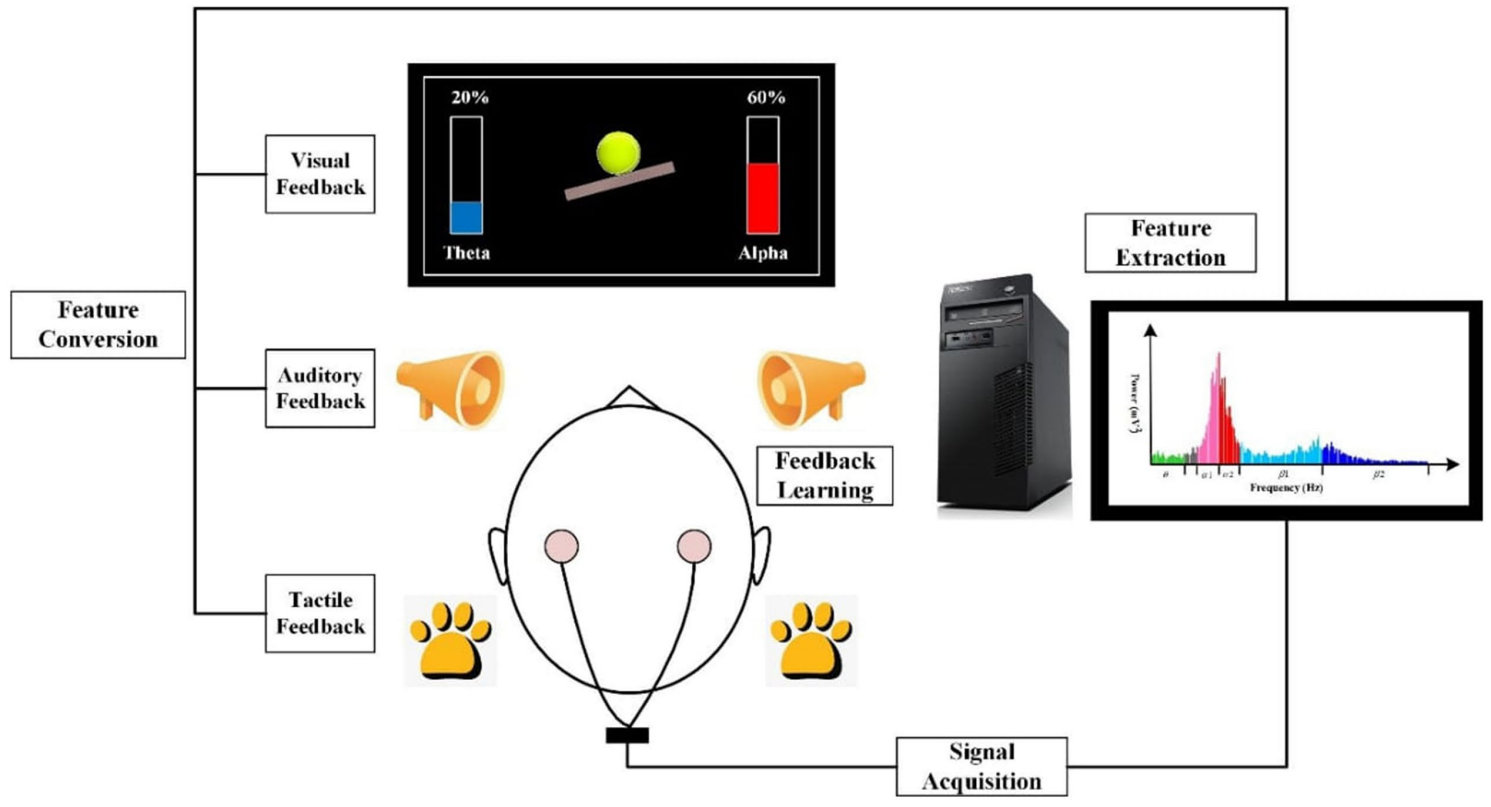
The principle of EEG neurofeedback. EEG neurofeedback (NFB) system typically consists of four stages of processing measured EEG signal: (1) Signal acquisition; (2) Feature extraction; (3) Feature conversion, and (4) Feedback learning. NFB is based on measuring an individual’s brain activity by means of an electronic instrument. The measured EEG signal is then analyzed and selectively converted into the form of a signal, which is easily perceived by the trained person. In case the target EEG activity is at least as high as the reward threshold, the participant receives feedback, which can be visual (for example, watching an ongoing graph of one’s own EEG activity that targets the NFB protocol), auditory or tactile. Learning and controlling the feedback signal provided by the NFB system leads to the enhancement of self-regulation of one’s brain activity which is essential for the goals of NFB treatment (Source: Produced with permission from Gong et al., 2021).

In addition to the external periodic application of electromagnetic fields through tACS, the EEG activity can also be non-invasively modulated by endogenous self-regulation of target EEG activity. EEG biofeedback or neurofeedback (NFB) represents a non-invasive neuromodulation method based on self-regulation of an individual’s own brain activity, which is achievable when external (auditory and/or visual) feedback is provided to the participant (Enriquez-Geppert et al., 2017; Othmer and Othmer, 2017). Imagine that we want to reward an increase in alpha activity (8–12 Hz), which plays a vital role in many brain processes, such as memory (Klimesch, 1999). Scalp EEG electrodes are positioned on the participant’s head, as soon as their current mental state aligns with the minimum value of the amplitude of EEG alpha activity, the participant receives auditory or visual feedback generated by the NFB software. Due to the consistent co-occurrence of external feedback and increased levels of desired EEG activity, the brain is capable of associating the instances wherein it receives rewards from external auditory and visual feedback with the states of increased EEG activity. Based on the principle of brain plasticity, it becomes easier and easier for the brain to produce the desired pattern of EEG activity (Gruzelier, 2014a; Enriquez-Geppert et al., 2017). Similar to tACS, both online and offline NFB effects modulate the amplitude of the target EEG activity (Bazanova et al., 2009; Neuling et al., 2012; Stecher et al., 2017; Deiber et al., 2020). Figure 1 illustrates the principle of neurofeedback.

Both tACS and NFB have been shown to modulate various EEG bandwidths (Neuling et al., 2013; Ketz et al., 2018; Pimenta et al., 2018), improve neurological conditions such as depression (Alexander et al., 2019; Lee et al., 2019), schizophrenia (Ahn et al., 2019; NFB), pain (Prim et al., 2019; Carlson and Ross, 2021), and improvement of brain functions including memory (Polanía et al., 2012; Lavy et al., 2019) and creativity (Agnoli et al., 2018; Luft et al., 2019). Since tACS and NFB, as two non-invasive neuromodulation techniques, operate according to fundamentally different principles, we hypothesize that the inclusion and mutual comparison of both methods could provide some fruitful insights into the field of research dealing with the functions and dynamics of the CFC. In this article, we discuss the current state of knowledge regarding CFC types and their functions, factors influencing CFC dynamics, and tACS and NFB protocols. Drawing from this knowledge, we suggest that tACS and NFB represent unique tools for the investigation and therapeutic intervention of brain functions and conditions associated with some specific CFC patterns.

## 5 Cross-frequency coupling in EEG

Cross-frequency coupling (CFC) refers to the biophysical and functional coupling between two or more EEG frequency bands that occurs at both corticocortical and thalamocortical levels (Vanneste et al., 2018). The mechanism of functional coupling between two or more distinct EEG frequencies is thought to occur at the neuronal and synaptic levels (Salimpour and Anderson, 2019). At the neuronal level, CFC is thought to reflect the electrical coupling between individual neurons. At the synaptic level, CFC is thought to be the result of communication between excitatory and inhibitory synapses (Salimpour and Anderson, 2019). Coupling between different EEG frequencies is believed to result from several possible biophysical mechanisms. The physical properties of EEG, including amplitude, phase, and frequency, play an essential role in enabling coupling between distinct EEG frequencies (Jirsa and Müller, 2013; Yeh et al., 2023).

In this article, we will discuss CFC types in terms of the coupling mode between CFC components and the occurrence of CFC types in tACS and NFB studies.

### 5.1 What is driving and what is responding element in CFC?

A couple of interesting questions that are addressed in CFC research are as follows: Which CFC component of a given CFC pair acts as the driving element and which CFC component serves as the responding element? By driving element, we mean the CFC frequency that drives the second CFC component that responds to the activity of the driving CFC component (Hyafil et al., 2015). It is generally assumed that the low-frequency CFC component drives the high-frequency CFC component (Schroeder and Lakatos, 2009; Canolty and Knight, 2010). Based on biophysical properties of the EEG spectrum, following are the two supporting arguments for this assumption: First, low-frequency EEG bandwidths (delta, theta) and mid-frequency (alpha) tend to have a more long-lasting presence in the EEG spectrum, in contrast to high-frequency EEG activity (beta, gamma), which tends to occur as intermittent phasic bursts (Buzsáki, 2006; Othmer and Othmer, 2017). Second, there is a pink noise-like distribution of the EEG spectrum. According to the pink noise-like distribution of the EEG spectrum, the lower the EEG frequency, the more global its occurrence in the brain and vice versa (Buzsáki, 2006; Othmer and Othmer, 2017). Despite these notions, a high CFC component can sometimes act as a driving frequency (Shi et al., 2019). To disentangle the question of which frequency is driving and which is the responding frequency in CFC pairs, Helfrich and colleagues conducted an experiment in which they tested the effect of 10 Hz (alpha) tACS and 40 Hz (gamma) tACS on alpha–gamma CFC (Helfrich et al., 2016).

The results of the study were as follows: Alpha tACS induced a phase–amplitude CFC in which gamma amplitude was preferentially locked to the troughs of alpha oscillations. On the contrary, gamma tACS led to a reduction of alpha amplitude through amplitude-envelope correlation (Helfrich et al., 2016). Aligned with these results, it becomes evident that both low- and high-CFC components can function as both driving and responding elements. One of the proposed hypotheses to explain this phenomenon is that different coupling modes between CFC components can guide opponent brain processes (Schroeder and Lakatos, 2009). More future studies are needed to discover the relationship between different coupling modes of a particular CFC pair linked to their brain functions.

### 5.2 Bidirectional vs. unidirectional coupling

Based on the study of (Hyafil et al., 2015), there are two different coupling modes: bidirectional and unidirectional coupling modes.

1. **Unidirectional coupling** refers to the situation when one frequency component (driving frequency) modulates the second (responding) CFC component without being reciprocally modulated by the responding CFC component (Hyafil et al., 2015). As a specific example of such a coupling mode, one could mention the situation when the tACS stimulation frequency alpha tACS modulated beta EEG activity (Berger et al., 2018). The enhancement of beta amplitude did not reciprocally influence the tACS stimulation frequency alpha. If the beta enhancement in this study resulted from alpha tACS, it can be considered as an unidirectional coupling between the driving frequency (alpha tACS frequency) and the responding frequency (beta EEG frequency).
2. **Bidirectional coupling** refers to the phenomenon whereby one CFC component can drive the first CFC component, and conversely, the second CFC component can drive the first CFC component (Hyafil et al., 2015). For example, the findings of the aforementioned study (Helfrich et al., 2016) demonstrated that both alpha tACS and gamma tACS can modulate the coupling between alpha and gamma (Helfrich et al., 2016). In this study, it is important to note that alpha and gamma tACS did not lead to identical coupling modes between alpha and gamma (Helfrich et al., 2016). Therefore, we propose a further classification of bidirectional coupling as follows: asymmetric and symmetric bidirectional couplings.
  - **Asymmetric bidirectional coupling**: We use this term to describe the situation where one CFC component drives the second CFC component, resulting in a different CFC mode between the CFC components. For example, in the study by Helfrich et al. (2016), alpha tACS led to phase–amplitude coupling between alpha and gamma, whereas gamma tACS induced alpha–gamma amplitude envelope correlation (Helfrich et al., 2016).
  - **Symmetric bidirectional coupling**: We use this term to describe the phenomenon where the first CFC component drives the second and vice versa, resulting in an identical coupling mode between the CFC components. For example, symmetric bidirectional coupling was observed in an NFB study comparing the effects of sensorimotor rhythm (SMR) and beta NFB protocols (Pimenta et al., 2018). In the experimental group that underwent sessions of the NFB protocol aimed at increasing the amplitude of beta (15–18 Hz), session-to-session increases were present not only in the range of beta activity but also in the adjacent sensorimotor rhythm (SMR; 12–15 Hz) in central brain areas (Pimenta et al., 2018). Surprisingly, increases in amplitude from session to session were also present in both beta and SMR bandwidths following SMR amplitude upregulation protocols (Pimenta et al., 2018).

Whether the cited example of bidirectional coupling between beta and SMR from Pimenta et al. (2018) was indeed the example of symmetric CFC between two independent EEG bandwidths may be debatable, as the selected bandwidths SMR (12–15 Hz) and beta (15–18 Hz) overlap. However, some evidence suggests that they are related to various brain functions and different brain states (Gruzelier, 2014a; Gruzelier, 2014b; Lee et al., 2013; Takahashi et al., 2011; Zhang et al., 2008) with their origin stemming from different brain areas (Arns et al., 2009). Beta is more central and frontal in the left hemisphere, whereas SMR is more central in the right hemisphere (Arns et al., 2009).

In terms of functions, SMR is believed to play a role in enhancing the recruitment of diverse neural functions associated with the regulation of brain processes (Pimenta et al., 2018). This aligns with the theory that lower EEG frequencies are responsible for regulating more global processes in the brain (Othmer and Othmer, 2017). Functions of beta appear to include cognitive processes such as top-down control of attention (Lee et al., 2013), response inhibition (Zhang et al., 2008), and cognitive task engagement (Takahashi et al., 2011). Differential effects of NFB training targeting the upregulation of SMR and beta have also been reported (Gruzelier, 2014b). While NFB-related beta enhancement was associated with greater arousal, and faster reaction times but greater fatigue immediately after NFB training, SMR NFB had a more calming effect (Gruzelier, 2014b). However, SMR and beta also appear to have overlapping functions. Positive correlations have been reported between motor inhibition and beta enhancement (Picazio et al., 2014) and between motor inhibition and SMR activity enhancement (Lubar and Shouse, 1976). Functional and frequency overlap may play an essential role in the symmetric bidirectional CFC between the investigated EEG bandwidths. This assertion might be supported by the results of Pimenta et al. (2018) study, in which NFB-related amplitude increases of SMR and beta overlapped bilaterally in central and parietal brain regions (Pimenta et al., 2018).

Having established that the coupling mode between two particular CFC components is bidirectional, we believe it is also worth investigating whether the coupling mode is symmetric or asymmetric. Such an investigation may also be of therapeutic importance, especially for people whose EEG frequency of interest does not respond to external brain modulation involving that particular EEG frequency. Let us take SMR as an example. For example, some individuals with excessively low resting SMR activity have been found to be unresponsive to the SMR NFB protocol (Weber et al., 2011). In such cases, modulation through other EEG frequencies that have symmetric bidirectional coupling relationships with SMR may enhance SMR amplitudes.

### 5.3 Challenges by investigating unidirectionality and bidirectionality of CFC

Although studying the directionality of CFC in the EEG may be a promising area of research, it is necessary to mention some methodological challenges. Let us assume that we want to apply tACS/NFB to enhance a particular CFC pair consisting of X and Y EEG components. Based on the previous definition of unidirectional and bidirectional CFC (Hyafil et al., 2015) and tACS and NFB studies that discovered bidirectional CFC (Hyafil et al., 2015; Pimenta et al., 2018), we would estimate that bidirectional CFC would enhance either the NFB/tACS frequency X EEG frequency X, and the enhanced X EEG frequency would drive the Y EEG frequency. Conversely, tACS with Y stimulation frequency would enhance the Y EEG frequency, driving its X EEG CFC component. However, there may not always be a straightforward relationship between NFB/tACS, target EEG frequency, and resulting CFC dynamics. An enhancement of the Y EEG frequency component by an enhancement of the X EEG frequency component during or after the application of NFB/tACS at the target X frequency might be due to other than functional (biological) relationships between X and Y EEG. For example, tACS with X stimulation frequency might stimulate Y EEG frequencies simply due to their biophysical properties via resonance principle, provided Y is an X integer (Veniero et al., 2015). Coupling between the X and Y components of CFC via NFB/tACS intervention with target X frequency may also be complicated by methodological difficulties associated with tACS, such as reduced reactivity to alpha tACS with eyes closed (Berger et al., 2018; Lefebvre et al., 2017). The lack of success in inducing unidirectional or bidirectional CFCs between X and Y EEG frequencies by applying NFB/tACS with target X frequency might be due to the contextual dependence of certain CFCs on a specific situation. For example, some EEG patterns are only present in active states, for example, during specific cognitive tasks (Rodriguez-Larios et al., 2020; Klimesch, 1999). Therefore, tACS must be applied during these specific conditions to improve the desired EEG pattern regarding brain function (Feurra et al., 2013). It is also necessary to differentiate between CFC patterns in some specific brain areas in resting and active states. For example, CFC between alpha and theta can occur in both resting and active states, and in each state, it represents an entirely different function (Rodriguez-Larios et al., 2020). Alpha–theta CFC was found to be present during rest, meditation, and arithmetic tasks (Rodriguez-Larios et al., 2020). Bidirectionality or unidirectionality was not tested in this study. However, alpha–theta CFC’s biophysical properties differed in each condition: Non-harmonic coupling was present in the resting condition. In contrast, harmonic coupling was present in the active conditions (Rodriguez-Larios et al., 2020). This mention is only to illustrate that the same CFC patterns may have different functions and biophysical properties under various conditions, it is crucial to consider this source of variability when investigating whether there is unidirectionality or bidirectionality in a specific CFC pattern.

### 5.4 Types of CFC patterns and their occurrence in tACS and NFB studies

**Figure 2:**
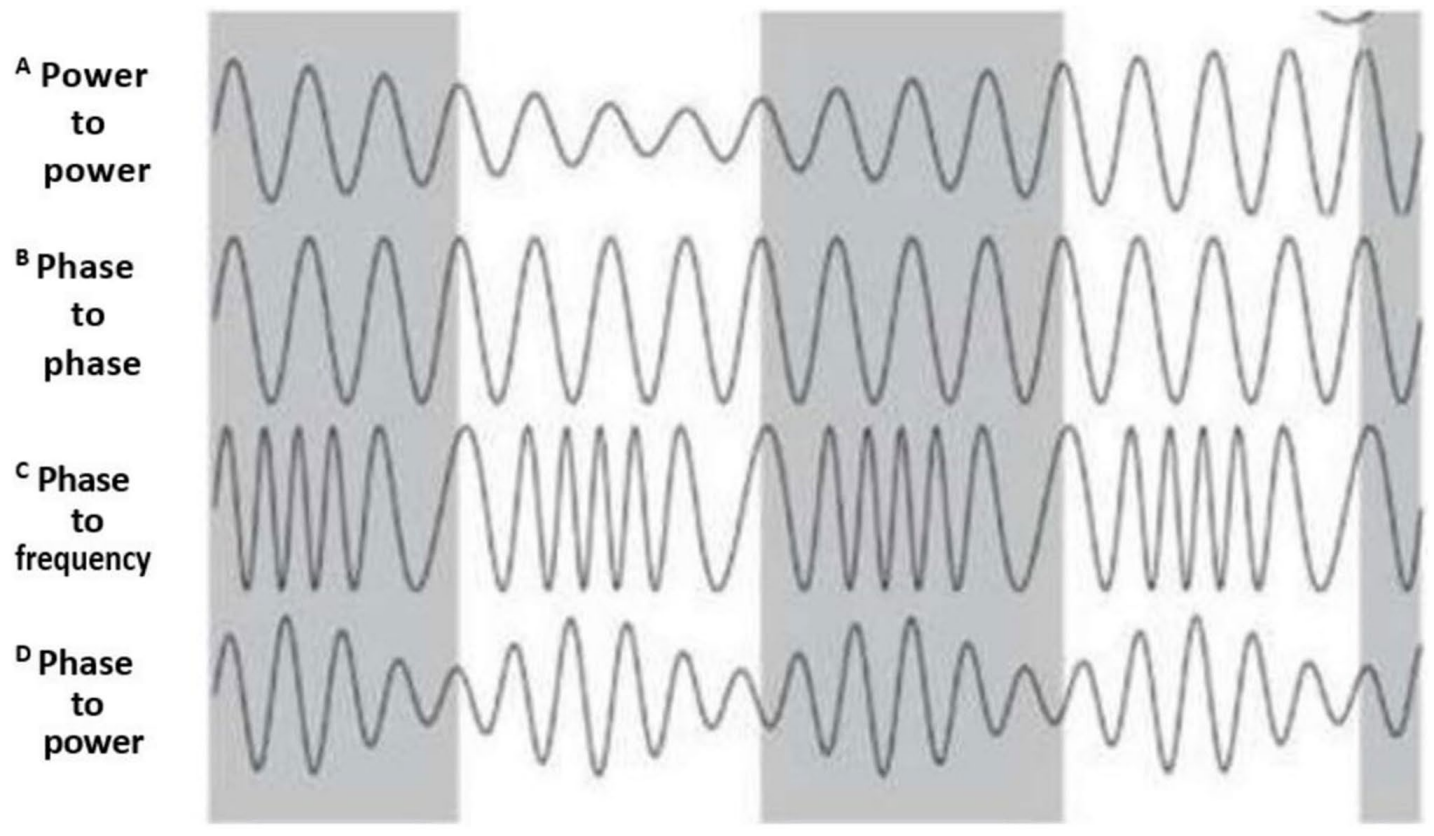
CFC types. The figure depicts CFC types according to the biophysical characteristics of coupling between CFC components. **(A)** Power–power (or amplitude–amplitude) CFC: amplitude of one EEG frequency drives the amplitude of another EEG frequency; **(B)** Phase– phase CFC: phase of one EEG frequency modulates the phase of another EEG frequency. **(C)** Phase-to-frequency CFC: phase of one EEG frequency modulates the frequency of another EEG frequency. **(D)** phase–amplitude CFC: phase of one EEG frequency modulates the amplitude of another EEG frequency. Gray color corresponds to one CFC component, and the white represents the second CFC component (Produced with the author’s permission from Pevzner et al., 2016). The legend originally used in this adopted figure from Pevzner et al. (2016) starts with B and ends with E.

Depending on which biophysical EEG characteristics are involved in CFC, there are several CFC types. The most common CFC types are (1) phase–amplitude coupling; (2) amplitude–amplitude coupling; (3) amplitude–frequency coupling; and (4) phase–phase coupling. To detect a specific trend in EEG dynamics, such as phase–amplitude coupling between two different EEG frequencies, the coupling between the phase and amplitudes of these frequencies must achieve statistical significance during the observation period (Hyafil, 2015; Shi et al., 2019). A comprehensive summary of each CFC type is presented in Figure 2.

#### 5.4.1 Phase–amplitude coupling

Phase–amplitude coupling (PAC) refers to a coupling mechanism between two frequencies in which the phase of one EEG frequency modulates the amplitude of another (Yeh et al., 2023; Jirsa and Müller, 2013).

PAC is probably the most studied type of CFC (Yeh et al., 2023; Hülsemann et al., 2019). This CFC type has been repeatedly reported to occur between different EEG frequencies during various brain functions, such as theta–gamma coupling during working memory performance (Lisman and Jensen, 2013; Biel et al., 2021), alpha–gamma CFC during visual representations (Jensen et al., 2014), alpha–theta PAC during meditation (Rodriguez-Larios et al., 2020), and arithmetic tasks (Rodriguez-Larios and Alaerts, 2019; Rodriguez-Larios et al., 2020).

To date, phase–amplitude CFC has been reported in a single-frequency tACS study, in which in-phase 40-Hz (gamma) tACS applied over parieto-occipital areas resulted in an increase in gamma synchronization that correlated with a reduction in alpha amplitude (Helfrich et al., 2014a). To the best of our knowledge, no NFB study has reported phase–amplitude CFC after the application of a single-frequency NFB protocol. However, it appears that the occurrence of phase–amplitude CFC has not yet to be explored in single-frequency NFB studies.

With regard to multi-frequency tACS studies, multifrequency tACS protocols designed in such a way that the application of one tACS is locked to the particular phase of another tACS frequency have been enormously exploited in recent years. On the contrary, the use of phase–amplitude NFB protocols appears to be a relatively new field of study. Nevertheless, the intentional induction of phase–amplitude NFB has been shown to be possible. One NFB study used a phase-amplitude CFC downregulation protocol between beta (13–30 Hz) and gamma (50–100 Hz) in the motor cortex, resulting in successful downregulation of this type of CFC (Izutsu et al., 2023).

Multifrequency tACS protocols are typically designed as follows: A low-frequency tACS component is permanently induced, and in the respective phase of the low-frequency tACS component, typically in the peaks or troughs, a high-frequency component is induced (Turi et al., 2020; Zhang et al., 2023). Roughly speaking, these types of tACS protocols are constructed according to the principle of phase–amplitude coupling since the induction of the high-frequency tACS component (non-zero value of its amplitude) depends on the particular phase of the low-frequency tonic tACS component. These types of tACS protocols have modulated brain functions, such as working memory (Turi et al., 2020; Zhang et al., 2023) and emotional regulation (Bramson et al., 2020).

#### 5.4.2 Amplitude–amplitude coupling

Amplitude–amplitude (AAC) CFC refers to the co-modulation of amplitudes of different EEG frequencies (Yeh et al., 2023; Hyafil et al., 2015) and typically occurs between two adjacent EEG frequency bandwidths, for example, between delta and theta. It has been found that ACC tends to be symmetric, i.e., modulation of amplitudes of one frequency results in an amplitude change of another EEG frequency and vice versa (Jirsa and Müller, 2013). There are two subtypes of AAC CFC: Positive AAC and negative AAC. Positive AAC reflects the situation when an increase in the amplitude of one EEG frequency is accompanied by an increase in the amplitude of another EEG frequency. On the contrary, negative AAC refers to the phenomenon when the increase in the amplitude of one EEG frequency bandwidth is positively correlated with a decrease in the amplitude of another EEG frequency (Hyafil et al., 2015). For example, during sleep, negative AAC occurs between lower EEG frequencies (theta, delta) and higher EEG frequencies (alpha, beta) in the following manner: Increases in theta and delta amplitudes are accompanied by decreases in alpha and beta amplitudes (Shi et al., 2019).

In single-frequency NFB studies, positive AAC is a relatively common phenomenon. To name a few examples, in the study by Pimenta et al. (2018), there was a simultaneous increase of sensorimotor rhythm (SMR; 12–15 Hz) and beta activity (15–18 Hz) in central brain areas during sessions in which beta amplitude upregulation protocol was used (Pimenta et al., 2018). Similarly, the beta amplitude upregulation protocol (12–22 Hz) resulted in a concomitant increase in alpha amplitudes (8–12 Hz; Jurewicz, 2018). Alpha upregulation protocols were found to increase not only alpha but also theta activity (Hanslmayr et al., 2005). Several tACS studies have documented positive ACC in single-frequency tACS studies. For example, applying 10 Hz (alpha tACS) resulted in an increase in alpha amplitudes that paralleled an increase in gamma EEG activity (Castellano et al., 2020). Another study found a positive correlation between alpha and beta amplitudes after administering alpha tACS (Alagapan et al., 2016).

Negative AAC has also been reported in some single-frequency NFB and tACS studies. In one NFB study, an alpha upregulation protocol increased alpha amplitude with a concomitant decrease in delta activity across NFB sessions (Nan et al., 2012). Another alpha upregulation protocol study found that decreased gamma amplitude correlated with increased alpha amplitude (Bagherzadeh et al., 2020). A similar trend was found after applying tACS with individualized alpha frequency (Herring et al., 2019).

Speaking of multifrequency protocols designed to drive the ACC selectively, the NFB has a long history of such protocols. Traditionally, simultaneous upregulation of SMR amplitude and simultaneous downregulation of theta amplitude have been successfully used to treat attention deficit hyperactivity disorder (Gruzelier, 2014a; Gruzelier, 2014b). A more complex multifrequency NFB protocol aimed at upregulation of alpha amplitude by simultaneous downregulation of beta and gamma activity was successfully used to treat tinnitus (Vanneste et al., 2016). On the contrary, to the best of our knowledge, ACC tACS has not yet been exploited in the field of multifrequency tACS protocols.

One of the possible functions of some specific ACC types could be the regulation of arousal. For example, tonic mental hyperarousal has been found to correlate with excessively high beta amplitudes and reduced low alpha amplitudes (Ko and Park, 2018), indicating a negative ACC relationship between alpha and high beta activities. NFB was found to reduce high beta and increase low alpha, which is associated with the reduction of hyperarousal (Ko and Park, 2018; Ancoli and Kamiya, 1978). To the best of our knowledge, no studies have been conducted to investigate the ability of tACS to modulate mental arousal state via modulation of low alpha and high beta activity. We believe investigating the relationship between arousal and alpha and high beta activity could yield fruitful results. In favor of our hypothesis, a positive correlation between high beta activity and salivary cortisol concentration has been found (Schutter and Knyazev, 2012; Díaz et al., 2019).

#### 5.4.3 Amplitude–frequency coupling

Amplitude–frequency coupling (AFC) refers to the mode of coupling in which amplitude changes at one EEG frequency are associated with frequency changes at another EEG frequency (Jirsa and Müller, 2013; Hyafil et al., 2015; Yeh et al., 2023). In one NFB study, a downregulation of alpha amplitude was positively associated with an increase in high mean gamma frequency (60–100 Hz; Ros et al., 2010). This study also found a positive correlation between the downregulation of alpha amplitude and cortical excitability as measured by motor-evoked potentials (MEPs) induced by repetitive transcranial magnetic stimulation (Ros et al., 2010). Although exploring the relationship between cortical excitability and gamma frequency is beyond the scope of this study, based on the antagonistic role between alpha and gamma activities in terms of activation vs. inhibition of brain areas (Spaak et al., 2012; Herrera et al., 2016), it may be important to investigate whether this tendency also applies to alpha–gamma AFC.

#### 5.4.4 Phase–phase coupling

Phase–phase coupling (PPC) is a type of coupling mode based on phase co-modulation of different EEG frequencies (Jirsa and Müller, 2013; Hyafil et al., 2015; Yeh et al., 2023), which is thought to enable neural communication between distant cortical regions through coherent communication (Darvas et al., 2009; Jirsa and Müller, 2013; González et al., 2020). For example, PPC between theta and gamma has been found in the occipital and frontal regions during sleep (Stankovski et al., 2017).

To our knowledge, this type of CFC has not yet been investigated in neurostimulation studies using different types of tACS and NFB protocols.

### 5.5 Primary and secondary CFC types

We propose that NFB and tACS may induce and/or influence two distinct categories of CFCs: We propose the terms (1) primary CFC and (2) secondary CFC.

**Primary CFC**: We define this category as that type of CFC whose one CFC component is equal to the tACS frequency/NFB-rewarded EEG frequency and/or its harmonics. According to our hypothesis, primary CFC arises from tACS-related entrainment or NFB-related enhancement of the CFC component whose frequency value corresponds to the selected tACS or NFB-rewarded frequency. The CFC component, whose modulation is related to tACS or NFB, then drives the second CFC component. To further explain this mechanism, imagine we want to enhance frontal alpha (8–12 Hz) activity by tACS and NFB. To increase alpha amplitudes by NFB, we will set a minimum microvolt threshold. Whenever the instantaneous value of the participant’s alpha amplitude reaches at least the minimum threshold, the participant will receive NFB auditory and visual signals, leading to an increase in alpha amplitude. Speaking of tACS, we will choose 10 Hz (mean alpha value) or adjust the tACS frequency with respect to the participant’s individual alpha bandwidth. Both tACS (Zaehle et al., 2010; Neuling et al., 2013; Ahn et al., 2019) and NFB studies consistently report the ability of tACS and NFB to increase alpha amplitudes (Hanslmayr et al., 2005; Escolano et al., 2014). Since alpha activity is involved in numerous brain functions in both resting and task-active states (Palva and Palva, 2007; Klimesch, 2012) and tends to couple with other brain EEG bands (Wang et al., 2019; Rodriguez-Larios et al., 2020) alpha theta alpha gamma, it is likely that NFB- or tACS-induced enhancement of alpha activity would be accompanied by coupling with other EEG bands resulting in primary CFC.

**Secondary CFC**: With respect to neuromodulation by tACS and NFB, those CFC types would be defined as secondary CFC types that do not arise due to direct tACS-dependent entrainment or NFB-induced enhancement of target brain activity. On the contrary, we define secondary CFC types as CFC patterns induced or influenced by the indirect effects of tACS and NFB. tACS and NFB are indirect effects other than tACS-related entrainment or NFB-modulated EEG target frequency.

Starting with NFB, it is necessary to realize that NFB training is a very complex and active process, where several neural networks are supposed to cooperate to enable the participant to modulate neural activity in the desired direction successfully. Sensory and auditory processes necessary to receive and decode auditory and visual NFB-relevant signals must properly cooperate with attentional neuronal networks and brain parts responsible for adjusting brain activity to corresponding rewarded patterns (Berger and Davelaar, 2018). Motivation to improve the brain state for better functioning via NFB plays an important role in NFB conditioning (Legarda et al., 2011; Berger and Davelaar, 2018). The coupling between frontal theta phase and parieto-occipital gamma amplitude was positively correlated with reward evaluation (Riddle et al., 2022).

Additionally, goal-oriented behavior was associated with PAC between frontal delta and beta activity (Riddle et al., 2021, 2022). Because both goal-oriented behavior and reward evaluation are considered to play an essential role in successful NFB conditioning (Legarda et al., 2011; Berger and Davelaar, 2018), it is possible that the aforementioned CFC types may also occur during NFB training. We postulate that reward-oriented brain behavior can be associated with specific CFC, might found its support in the documented effects of dopamine on CFC modulation (Andino-Pavlovsky et al., 2017) since dopamine is significantly involved in rewards neuronal circuits (O’Doherty, 2004).

In summary, the NFB may induce CFC types outside the NFB-rewarded frequency and/or at the same frequency as the target frequency for NFB reward but via mechanisms other than direct NFB-reward-dependent enhancement. Such secondary CFC types may represent general features of neural dynamics associated with NFB conditioning independent of the selected NFB-rewarded protocol frequency.

In the context of tACS, we hypothesize that secondary CFCs may occur due to other effects of tACS on the cortex other than entrainment mechanisms. This category of “other effects of tACS” might include sensory processes associated with the processing of tACS-induced sensations, such as itching, tingling, pain, and burning sensations that have been documented in numerous tACS studies (Turi et al., 2013; Raco et al., 2014; Kvašňák, 2019). Our hypothesis could be supported by various CFC patterns related to sensory processing (Liu et al., 2015; Wang et al., 2019). For example, CFC between low gamma (40–70 Hz), high gamma (70–110 Hz), and phase of alpha activity in the frontal lobe, amygdala, and hippocampus have been identified during pain processing (Liu et al., 2015). Increased alpha in parieto-occipital regions and alpha–gamma phase–amplitude CFC have been strongly proposed to be associated with phosphene perception (De Graaf et al., 2017; Wagner et al., 2019). The integration of sensory signals from various modalities has been associated with coupling between alpha, beta, and theta rhythms in several brain regions, including sensorimotor, parietal, supramarginal, and midcingulate gyri (Wang et al., 2019). Based on these findings, the sensory effects of tACS may result in the emergence of specific CFC patterns.

For this reason, we believe that one must consider both primary and secondary CFC types when applying non-invasive neuromodulation, especially in cases where the CFC bandwidth of interest, used as the NFB reward frequency or tACS frequency, is involved in both primary and secondary CFC types.

#### 5.5.1 Our proposed methodological approach to recognize secondary CFC by tACS and NFB

To differentiate which CFC patterns are primary and which are secondary to NFB training, we would suggest conducting within-subject and between-subject studies comparing NFB conditioning of different EEG frequencies in different brain regions (EEG maps) with placebo conditions. Provided that some typical CFC pattern(s) would be universally present in several NFB protocols focused on training different EEG frequencies in different NFB regions and that these CFC patterns would not be significantly present in the placebo condition, it could be assumed that some specific (secondary) CFC patterns are inducible by different NFB protocols and that they might tell us something about universal brain dynamics associated with NFB training. Quantitative EEG analysis could be a valuable tool for analyzing EEG activity during and after NFB training in different brain areas. EEG quantitative analysis is based on EEG spectral analysis of different EEG bandwidths in different brain regions, significance probability mapping, and other analytical techniques. Quantitative EEG analysis can be performed on spontaneous EEG in various brain states or alongside sensory stimulation (Nuwer, 1988).

To identify potential CFCs associated with tACS, it is necessary to be familiar with sensory phenomena such as itching, tingling, pain, burning, and phosphenes (Kanai et al., 2010; Turi et al., 2013; Raco et al., 2014; Kvašňák, 2019; Kvašňák et al., 2022) that tACS can induce. Furthermore, we recommend studying CFC patterns that are directly related to the processing of the aforementioned sensory phenomena that can be induced by tACS. To our knowledge, the relationship between the subjective perception of tACS-induced sensory effects and the corresponding patterns has not been studied. To this end, we proposed the application of different tACS frequencies and intensities over specific brain regions known to be involved in sensory processing and simultaneously record tACS to capture online EEG activity in the brain, which is performed when investigating the immediate effect of tACS on ongoing EEG activity (Herrmann et al., 2016). EEG data will be correlated with subjective reports of tACS-induced sensory phenomena. Standardized questionnaires for measuring side effects of tACS, such as the Symptoms Self-Report Questionnaire, could be used for this purpose (Bjekić et al., 2024; Antal et al., 2017). Statistical analysis would be required to correlate changes in CFC with the intensity of perceived sensory effects of tACS between active tACS and placebo tACS groups.

## 6 Single-frequency and multifrequency tACS and NFB protocols

Both tACS and NFB studies include both single- and multifrequency protocols.

### 6.1 Single-frequency tACS and NFB protocols

In the field of tACS protocols, single-frequency protocols consist of the selection of a single stimulation frequency (Herrmann et al., 2013) or NFB frequency bandwidth (Gruzelier, 2014a), for instance, 10 Hz, corresponding to alpha activity (Klimesch, 1999), when we are interested in enhancing the amplitudes and/or coherence of alpha EEG activity.

Similarly, a number of NFB protocols are based on the upregulation of a single EEG bandwidth, for example, alpha (8–12 Hz; Hanslmayr et al., 2005; Escolano et al., 2014). In addition to single-frequency tACS protocols (Feurra et al., 2013; Wischnewski et al., 2019), single-frequency NFB protocols have also been shown to be effective in improving the amplitude and coherence of target EEG activity (van et al., 2012; Pimenta et al., 2018; Dessy et al., 2020). An important difference between single-frequency tACS and the NFB protocol is that, while NFB protocols can selectively up- and down-regulate target EEG activity (typically amplitudes), tACS cannot determine in advance whether target EEG activity should be up- or downregulated. Although many single-frequency tACS protocols have led to a selective enhancement of specific EEG activity (Neuling et al., 2013; Helfrich et al., 2014b; Ketz et al., 2018), some studies report a reduction in amplitudes of target EEG frequencies (Garside et al., 2015; Alexander et al., 2019; Zarubin et al., 2020). However, it is possible to employ tACS protocols where the stimulation frequency is applied in phase with the endogenous brain activity of the same frequency, thereby increasing the probability of enhancing entrained EEG activity. Conversely, anti-phase tACS can be utilized to diminish target EEG activity through phase interference (Helfrich et al., 2014a).

We believe that both NFB and tACS single-frequency protocols have advantages and disadvantages. Speaking of NFB, the advantage of being able to selectively decide whether to up- or downregulate the particular EEG bandwidth of interest is twofold: First, the ability of the subject to successfully selectively up- or downregulate the desired EEG activity may be of considerable therapeutic importance, telling us whether the subject is responding to treatment and what specific behavioral and neurophysiological effects the particular NFB protocol is exerting. Second, regarding the question of different EEG bandwidths involved in some specific CFC patterns consisting of high and low-frequency CFC components, NFB protocols designed to selectively up- or downregulate EEG activity having the same frequency spectrum as the high or low-frequency component of the CFC might provide answers to the questions: What happens to the low-frequency CFC component when the high-frequency CFC component is up- or downregulated? The disadvantage of NFB protocols is that, unlike tACS, NFB is based on active self-regulation of one’s own EEG activity. In contrast, tACS application is a relatively passive process for participants when it comes to attention allocation. Compared to tACS, the NFB procedure requires much attention from the participant to focus on finding and maintaining a mental state that rewards the target EEG activity. For this reason, the application of NFB would be more problematic during active cognitive and behavioral experimental tasks, during which numerous CFC patterns occur that are associated with the respective brain functions, such as memory (Popov et al., 2018; Biel et al., 2021; theta, alpha, and gamma) and emotional regulation (Bramson et al., 2020).

Despite working on fundamentally different principles, an effect common to tACS and NFB has been observed: In the study by Alagapan et al. (2016), alpha tACS was used to stimulate the alpha EEG bandwidth (Alagapan et al., 2016). In one participant, in whom the most significant increases in alpha amplitudes were present around 8 Hz, there was a significant increase at 16 Hz, corresponding to beta activity (Alagapan et al., 2016). This phenomenon is consistent with the resonance theory, which consists of the fundamental principle that the external driving frequency increases not only the amplitudes of the frequency of the system with the same value but also the frequencies whose value is equal to the multiplication by a positive integer value of the fundamental frequency - the so-called harmonics (Wang, 2010; Musall et al., 2014). A similar phenomenon was observed during the protocol of alpha amplitude upregulation, where not only the alpha amplitudes but also a remarkable increase of the beta EEG spectrum with a frequency about twice as large as the target alpha NFB reward bandwidth (8–12 Hz) occurred (Fell et al., 2002). Based on these findings, it can be assumed that NFB and tACS protocols can be used to study these CFC types, whose low- and high-frequency components are functionally and biophysically interlocked based on harmonic interrelationships.

### 6.2 Multifrequency tACS and NFB protocols

In contrast to single-frequency NFB and tACS protocols, multifrequency protocols are based on the simultaneous manipulation of two or more different frequencies. To date, several types of multifrequency protocols have been used.

In the field of NFB, simultaneous amplitude upregulation of one EEG frequency and downregulation of another EEG frequency band have been used (Gruzelier, 2014a; Vanneste et al., 2016). The relatively new NFB protocol also allows the co-modulation of amplitude and phase of two different EEG frequencies (Izutsu et al., 2023).

Multifrequency tACS protocols have been invented in the following fashions: Low-frequency tACS represents the tonic component of multifrequency tACS, and high-frequency tACS is periodically simultaneously co-induced in the particular phase of tonic low-frequency tACS. Typically, high-frequency tACS is induced in the troughs or peaks of low-frequency tACS amplitudes (Turi et al., 2020; Zhang et al., 2023).

### 6.3 Possible application of single- and multifrequency tACS and NFB protocols in cross-frequency coupling research

We speculate that single- and multifrequency tACS and NFB protocols may represent promising tools for investigating functions and neurophysiological mechanisms beyond EEG cross-frequency coupling interactions.

We suggest that multifrequency tACS and NFB protocols may be suitable candidates for investigating behavioral and neurophysiological effects of the particular CFC pattern as a whole, whose electrophysiological features and functions are already known and may also be of therapeutic relevance. For example, the phase–amplitude CFC between sleep slow oscillations and sleep spindles during slow-wave sleep represents an important neurophysiological mechanism for sleep-dependent declarative memory consolidation (Klinzing et al., 2016). Single-frequency neurostimulation methods, such as acoustic stimulation (Ngo et al., 2013) and tACS (Ketz et al., 2018), have already been used to study the effect of external acoustic or electrical periodic application of low frequency corresponding to the frequency band of sleep slow oscillations to investigate its effect on sleep architecture and memory performance. The external frequency used in the protocols was repeatedly found to enhance not only the low-frequency component with corresponding frequency (sleep slow oscillations) associated with improvements in declarative memory performance but also the occurrence of the high-frequency component (sleep spindles; Ngo et al., 2013; Ketz et al., 2018). These findings indicate a consistent functional and electrophysiological link between sleep slow oscillations and sleep spindles. They raise the question of whether simultaneous application of tACS, corresponding to the EEG frequency bands of sleep slow oscillations and sleep spindles, could result in enhanced CFC between these two EEG bandwidths and improved declarative memory performance.

On the contrary, single-frequency NFB and tACS protocols may be suitable candidates for investigating the dynamics and relationships between low- and high-frequency components of the respective CFC patterns. To illustrate this idea with a concrete example, we can again use the aforementioned example of CFC between sleep slow oscillations and sleep spindles. Since this type of CFC occurs during sleep when conscious self-regulation of EEG activity—requiring a waking state—is not possible, we will use this example to discuss the possible application of the tACS single-frequency protocol. External modulation of slow sleep oscillations (low-frequency CFC component) or sleep spindles (high-frequency CFC component) by application of tACS with corresponding frequencies could potentially allow us to find out whether external modulation of low tACS frequency will modulate the occurrence of slow sleep oscillations and whether tACS induced modulation of slow sleep oscillations will correlate with modulation of occurrence of sleep spindles. On the contrary, it can also be investigated whether tACS-induced modulation of sleep spindles will have an effect on CFC interaction with slow sleep oscillations and whether the types of CFC interactions will differ from each other when tACS with a frequency corresponding to slow sleep oscillations and tACS frequency corresponding to sleep spindles are used.

Speaking of this particular CFC between sleep slow oscillations and sleep spindles, there is already ample evidence that external enhancement of sleep slow oscillations during NREM sleep leads to increased occurrence of sleep spindles (Ngo et al., 2013; Ketz et al., 2018) but other EEG bandwidths that are functionally and biophysically related to slow oscillations (e.g., harmonics of sleep slow oscillations at higher EEG frequencies) may also be influenced by some specific CFC patterns during external enhancement of sleep slow oscillations. There are several documented tACS and NFB single-frequency studies in which various forms of CFC between the target EEG frequency and other EEG frequencies were documented (Jurewicz, 2018; Pimenta et al., 2018; Herring et al., 2019; Castellano et al., 2020).

We hypothesize that single-frequency neuromodulation methods could be a suitable candidate for studying driving vs. responding frequency elements in CFC and the type of coupling mode between CFC components in terms of its type (e.g., phase–amplitude), directionality (unidirectional vs. bidirectional), and for bidirectional CFC types, whether the bidirectionality is symmetric or asymmetric.

Our proposed perspectives on the applicability of single- and multifrequency tACS and NFB protocols in CFC research are summarized in Table 1.

**Table 1:** Proposed applications of single and multifrequency tACS and NFB protocols in CFC research.

Single-frequency tACS and NFB protocols	Multifrequency tACS and NFB protocols
Directionality of CFC	Therapeutic and research use of tACS and NFB to modulate CFC patterns with known functions and brain locations.
Driving vs. responding frequency elements in CFC	
In how many CFC interactions with other EEG bandwidths participate in one particular EEG frequency?	

## 7 Possible challenges in studying CFC—sources of variability in CFC

Some pitfalls need to be mentioned when it comes to possible challenges associated with studying CFC using non-invasive brain methods, that is, there are many sources of variability in CFC patterns, such as EC vs. EO condition (Jirsa and Müller, 2013; Stankovski et al., 2017), resting vs. task-active condition (Jiang et al., 2015; Rodriguez-Larios et al., 2020), motivation (Riddle et al., 2022), age (Knyazev et al., 2019; Liang et al., 2021), experience (He et al., 2008), personality traits (Knyazev et al., 2019), fatigue (Liu et al., 2023), health (de la Salle et al., 2024; Ibrahim et al., 2014), and duration of occurrence of CFC patterns (Schutter and Knyazev, 2012).

EC vs. EO state was found to influence the directionality and strength of CFCs (Jirsa and Müller, 2013; Stankovski et al., 2017). CFC strength was repeatedly found to be higher in EC than EO (Jirsa and Müller, 2013; Stankovski et al., 2017). Furthermore, some CFC types tend to be more pronounced in EC, as was found for alpha–delta coupling (Jirsa and Müller, 2013). For this reason, we believe that it is necessary to precisely determine whether tACS or NFB intervention would take place in EC or EO conditions, and special attention should be paid to the detection and recognition of EEG artifacts caused by eye blinks (Noureddin et al., 2012).

Resting and active-task states also have a tremendous impact on CFC dynamics, such as the mean frequency of CFC components (Rodriguez-Larios et al., 2020), the directionality of CFC patterns (Jiang et al., 2015), and the biophysical coupling modes between CFC components (Rodriguez-Larios et al., 2020). For example, it has been shown that the coupling between alpha and theta is harmonic during mathematical tasks (active-task state). In contrast, the coupling mode becomes non-harmonic during resting (Rodriguez-Larios et al., 2020). Regarding the applicability of NFB and tACS, tACS is a better tool than NFB when studying potential differences in CFC between resting and task-active states. In comparison to NFB, where the participant must actively self-regulate their mental state to receive NFB-related external rewards, the external application of tACS is a relatively passive phenomenon for a participant.

Motivation is another factor that may have a significant impact on CFC, as specific patterns of CFC have been found to correlate with increased motivation and goal-directed behavior (Riddle et al., 2022). Motivation to participate in experiments can vary. Reasons for participation are boredom, enthusiasm for science, fun, or interest in self-learning (Jun et al., 2017). These types of motivation can influence the choice of study, attention, and dropout. For example, boredom was found to affect the level of attention negatively (Jun et al., 2017). Since attention is directly related to some specific CFC patterns, such as theta–gamma CFC, which is associated with increased attention (Sauseng et al., 2008), a significant decrease in attention due to lack of motivation on the part of participants could likely lead to a change or reduction in CFC patterns associated with attentional processes. Based on these findings, we propose that different motivation levels to participate in the experiment could cause inter- and intraindividual differences in EEG activity. Pre- and postexperiment questionnaires on subjective motivation could be particularly important in this regard.

Similarly, personality trait questionnaires should be used due to existing differences in CFC patterns between introverts and extroverts (Knyazev et al., 2019).

The duration of the NFB and tACS intervention and the difficulty of the experimental behavioral tasks, should be sufficient to avoid causing significant levels of fatigue among participants. It has been found that fatigue is associated with increased beta–gamma CFC in frontal and parietal regions (Liu et al., 2023), which could be of great importance when studying beta–gamma CFC related to functions other than fatigue in these brain areas.

The selection of a specific age group is also important. The strength of CFC and the occurrence of some CFC patterns differ between adults and children (Knyazev et al., 2019; Liang et al., 2021), which might be caused by the lifetime reorganization of neural networks (Müller and Lindenberger, 2012). Experience is also another factor that plays an important role in shaping CFC patterns (He et al., 2008).

Health status is another important consideration when studying CFC patterns. Some psychiatric and neurological conditions are associated with specific CFC tendencies (Ibrahim et al., 2014; de la Salle et al., 2024).

Last but not least, the duration of occurrence of the investigated CFC patterns should also be considered. Some CFC patterns have been found to have a short-term, transient occurrence. In contrast, other CFC patterns have a somewhat longer duration and are thought to reflect somewhat more stable processes, such as emotional states or personality traits (Knyazev, 2011; Schutter and Knyazev, 2012).

### 7.1 Proposed methodological approaches investigation or reduction of the proposed potential inter- and intraindividual sources of variabilities in CFC patterns

We propose methodological approaches to investigate or reduce potential intra- and interindividual sources of variability in CFC patterns.

#### 7.1.1 Selection of a specific demographic group

Selection of some particular demographic groups regarding factors such as health status and age of participants could help eliminate sources of variability in CFC patterns in different demographic groups (Knyazev et al., 2019; Liang et al., 2021). On the contrary, deliberately selecting two different demographic groups by studying a specific CFC pattern in a specific condition could be an interesting area of research.

#### 7.1.2 Determination of the experimental condition

Determination of the experimental condition in terms of EEG measurements during active vs. resting condition, as well as selection of EC vs. EO state, could further help to reduce potential sources of variability in CFC patterns occurring in each condition (Jirsa and Müller, 2013; Stankovski et al., 2017; Rodriguez-Larios et al., 2020).

#### 7.1.3 Pre- and postsession assessment of fatigue levels

Parietal and frontal beta–gamma CFC correlated with fatigue (Liu et al., 2023) may represent the significant noise when examining CFC patterns consisting of beta and gamma frequency components in frontal and parietal regions related to other brain functions. For this reason, we propose that pre- and postsession assessment of fatigue levels in participants and subsequent correlation analysis with the occurrence of fatigue-related beta–gamma CFC patterns might help shed more light on the dynamics and occurrence of beta–gamma fatigue-related CFC patterns. It might also help to optimize experimental sessions to minimize fatigue related to the experimental procedure. Standardized medical questionnaires The Fatigue State Questionnaire, which aims to assess immediate fatigue levels, might be a practical measurement tool (Greenberg et al., 2016).

#### 7.1.4 Assessment of participants’ motivation to participate in experiments

To evaluate the level of motivation of participants to take part in experiments, we propose to include a standardized questionnaire in the experimental procedure. Intrinsic Motivation Inventory, which includes measures of interest/enjoyment, perceived competence and effort, value of usefulness, perceived pressure, and perceived choice (McAuley et al., 1989).

#### 7.1.5 Differentiation between transient vs. stable CFC patterns of investigated EEG frequencies

We postulate that it might be important to consider that different dynamics of investigated CFC patterns consisting of two or more different EEG frequencies might be related to functionally different processes (Knyazev, 2011; Schutter and Knyazev, 2012). An analysis of whether the durations of CFC patterns are statistically comparable across participants might be relevant in distinguishing between transient and more stable forms of the studied CFC patterns. Such an analysis might be helpful in cases where there are more possible CFC patterns consisting of two or more EEG frequencies of our research interest, and transient or stable temporary occurrence of these CFC patterns might point to their different brain functions (Knyazev, 2011; Schutter and Knyazev, 2012).

## 8 Results

Based on research, both tACS and NFB studies implement single-frequency and multifrequency protocols. Compared to tACS, NFB has a long history.

In terms of CFC types, both tACS (Zhang et al., 2023; Bramson et al., 2020; Herring et al., 2019) and NFB studies (Izutsu et al., 2023; Jurewicz, 2018; Pimenta et al., 2018) report occurrences of phase–amplitude CFC and amplitude-to-amplitude CFC. Based on our findings, the occurrence of phase–phase and amplitude-to-amplitude CFC has not yet been directly investigated in tACS and NFB studies. However, there is one documented NFB study observed amplitude-to-amplitude CFC between alpha and gamma (Ros et al., 2010).

Bidirectionality vs. unidirectionality of CFC has been previously investigated in tACS studies (Helfrich et al., 2016), while it has not yet been investigated in the NFB field.

Various factors, such as resting vs. task-active state (Jiang et al., 2015; Rodriguez-Larios et al., 2020), health state (de la Salle et al., 2024), motivation (Ibrahim et al., 2014; Riddle et al., 2022), EC vs. EO condition (Jirsa and Müller, 2013; Stankovski et al., 2017), experience (He et al., 2008), and duration of occurrence of CFC patterns (Schutter and Knyazev, 2012) were found to be able to influence CFC patterns.

Regarding the sources of variability in CFC, studies dedicated to the investigation of the following were found: age (Knyazev et al., 2019; Liang et al., 2021), personality traits (Knyazev et al., 2019), fatigue (Liu et al., 2023).

## 9 Discussion

This paper discusses the CFC types and possible ways of using tACS and NFB to study CFCs in the EEG. The following research directions have been discussed: (1) Applicability of single-frequency and multifrequency NFB and tACS protocols in CFC research; (2) proposed new CFC types related to tACS and NFB; and (3) factors contributing to the diversity of inter- and intra-individual CFC dynamics.

### 9.1 Applicability of single-frequency and multifrequency NFB protocols in CFC research

We suggest that both single-frequency and multifrequency tACS and NFB protocols can be used in CFC research. In our opinion, single-frequency tACS and NFB protocols represent a good option when it comes to studying their influence on those CFC patterns whose frequencies of at least one of the CFC components correspond to the NFB reward frequency or tACS frequency. On the contrary, multifrequency tACS and NFB protocols could be a useful tool when applied to study the dynamics and functions of already known CFC patterns in specific brain areas, for example, frontoparietal theta–gamma PAC is known to be involved in working memory functions (Biel et al., 2021). In the field of NFB, a variety of multifrequency protocols have also been used therapeutically (Gruzelier, Enriquez). From a therapeutic point of view, single-frequency NFB and tACS protocols may represent suitable candidates for modulating those CFC patterns that have at least one component responsive to NFB and tACS. In the case of a particular CFC pattern, e.g., alpha–theta PAC, if for some reason theta is not responsive to theta tACS/NFB but alpha is responsive to alpha tACS/NFB, then it can be assumed that alpha modulation at the appropriate alpha tACS or NFB frequency could enhance theta activity via the natural coupling between alpha and theta in the particular CFC alpha–theta pattern.

### 9.2 CFC types

We proposed a new classification of CFC patterns based on (1) CFC symmetry and directionality, and (2) whether the occurrence depends on the primary or secondary effect of NFB and tACS. According to Hyafil et al. (2015), CFC patterns are either unidirectional (where one CFC element always drives the second CFC element) or bidirectional (both CFC elements can act as both driving and responding elements; Hyafil et al., 2015). We proposed that bidirectional CFC patterns can be further divided into the following two subcategories: Symmetric and asymmetric CFC patterns. Symmetric coupling (with X and Y CFC components) means that both (X as the driving element, Y as the responding element) result in an identical coupling mode (e.g., phase–amplitude coupling) even in cases where Y is the driving element and X is the responding element. Conversely, if the coupling modes are different when X is the driving element and then Y is the responding element, and vice versa, it can be said that it is a bidirectional asymmetric coupling. We believe that symmetric and asymmetric coupling should be considered because different coupling modes between the CFC components of the same CFC pattern may represent various brain processes (Rodriguez-Larios et al., 2020). Speaking of primary and secondary CFC types, this proposed classification is based on the direct and indirect effects of tACS and NFB. Primary CFC types are defined as those resulting from effects of tACS and NFB related to modulation of the target frequency, which corresponds to a CFC component of the CFC pattern via the NFB reward principle or tACS-induced entrainment. On the contrary, indirect effects of tACS and NFB include phenomena such as tACS-related sensations (itching, tingling, pain) or NFB-associated mental states representing reward-oriented behavior (anticipation of auditory and visual NFB-related feedback), which are likely related to specific CFC patterns, namely, secondary CFC patterns (Riddle et al., 2022; Wang et al., 2019).

### 9.3 Factors contributing to variety in CFC dynamics

There are several factors that can cause significant variety in CFC behavior, and, therefore, they should be considered when studying CFC: motivation (Riddle et al., 2022) age (Liang et al., 2021; Knyazev et al., 2019) fatigue (Liu et al., 2023), experience (He et al., 2008), personality traits (Knyazev et al., 2019), health status (de la Salle et al., 2024; Ibrahim et al., 2014), EO state (Stankovski et al., 2017; Jirsa and Müller, 2013), and duration of occurrence of CFC patterns (Schutter and Knyazev, 2012), resting vs. task-active state (Jiang et al., 2015; Rodriguez-Larios et al., 2020).

To eliminate or at least reduce the unwanted effects of these variability factors, some steps can be taken, such as defining resting or task-active experimental conditions, as well as EO vs. EC conditions, and selecting a specific demographic group with respect to age and health status of the participants. Other steps may involve optimizing the duration and difficulty of the experimental intervention to prevent fatigue among participants and using questionnaires to assess factors such as personality traits and motivation levels of participants to participate in the study.

In conclusion, we believe that this summary of the state of the art regarding CFC, as well as the proposed research directions, could contribute to a deeper understanding of CFC and the effects of tACS and NFB on brain functions.

## Data Availability

The original contributions presented in the study are included in the article/supplementary material, further inquiries can be directed to the corresponding author.
